# Development and content validation of a mobile application for monitoring latent tuberculosis treatment

**DOI:** 10.1590/0037-8682-0465-2021

**Published:** 2022-03-25

**Authors:** Marcelle Temporim Novaes, Thiago Nascimento do Prado, Jessica Cristina Silva Delcarro, Silvia das Dores Rissino, Nathalia Yukie Crepaldi, Tiago Lara Michelin Sanches, Thomaz Felipe Soares Arnizant, Domingos Alves, Ethel Leonor Noia Maciel

**Affiliations:** 1 Universidade Federal do Espírito Santo, Laboratório de Epidemiologia, Vitória, ES, Brasil.; 2 Universidade Federal do Espírito Santo, Departamento de Enfermagem, Vitória, ES, Brasil.; 3 Universidade Federal do Espírito Santo, Departamento de Computação e Eletrônica, São Mateus, ES, Brasil.; 4 Universidade de São Paulo, Centro de Informação e Informática em Saúde, Ribeirão Preto, SP, Brasil.; 5 Instituto Federal do Triângulo Mineiro, Departamento de Tecnologia da Informação e Comunicação, Uberaba, MG, Brasil.; 6 Universidade de São Paulo, Departamento de Medicina Social, Ribeirão Preto, SP, Brasil.

**Keywords:** Validation studies, Mobile health, Latent tuberculosis

## Abstract

**Background:**

Non-compliance with latent tuberculosis infection (LTBI) treatment is a reality. The objective of this study was to develop and validate an mobile device application for monitoring the treatment of LTBI.

**Methods:**

We defined the requirements, elaborated on the application's conceptual map, generated implementation and prototyping alternatives, and validated content.

**Results:**

Feedback on the validity of content were: “usefulness, consistency, clarity, objectivity, vocabulary, and precision” from professionals, and “clarity” from patients.

**Conclusions:**

The application proved to be easy to understand, according to the assessment of both professionals and people undergoing treatment for LTBI.

According to the World Health Organization (WHO)^1^, nearly 10 million people developed tuberculosis (TB) and 1.4 million individuals died of tuberculosis infection worldwide in 2019; in Brazil, 73,864 novel cases of TB were diagnosed in that same year^2^. 

It is estimated that a quarter of the world’s population is infected with *Mycobacterium tuberculosis*, since most do not have signs and/or symptoms of the disease, a characteristic feature of LTBI. Progression to active TB depends on external factors, especially the integrity of the immune system^3^.

In Brazil, the treatment of LTBI is provided by the Unified Health System (*Sistema Único de Saúde*). The treatment lasts from 6 to 9 months, and isoniazid is the drug of choice, reducing the risk of illness due to active TB from 90% to 60%².

However, abandoning or not initiating LTBI treatment can be related to factors such as age, concern about the adverse effects of the medication, schooling, negative opinion provided by the specialist, and even discrimination^4^ ^-^ ^6^.

Therefore, with the objective of improving the adherence of patients with LTBI and consequently reducing the number of treatment abandonments, this study developed and validated a mobile application for monitoring the treatment of LTBI.

This is a methodological study aimed at creating an application for mobile devices that assists in the treatment of patients with LTBI, carried out in three stages: 1) definition of requirements, 2) prototyping alternatives, and 3) content validation^7^.

An application called “*Meu tratamento tuberculose latente*” (“My latent tuberculosis treatment”) was developed using the Android operating system. The images used were created by the study team using the Freepik tool.

In the first stage, a search was conducted in the Manual of Recommendations for the Control of Tuberculosis in Brazil and in the Protocol for Surveillance of Latent Infection by *Mycobacterium tuberculosis* in Brazil^8^ on the prevention and treatment of LTBI. These resources helped in the development of application content.

In the second stage, application prototyping was performed to understand the user's requirements. The next stage is the representation of the software visible to the user.

In the third stage, the content of the application was validated by health professionals and patients. For this, the study by Pasquali^9^ was used, which shows that content validation needs to be done by means of two analyses: analysis by judges (professionals) and semantic analysis (target audience). It is recommended that content analysis, as well as the inclusion criteria, be carried out by experienced people in the area in question, suggesting six to 20 subjects, requiring at least three individuals in each selected group^9^. To avoid possible ties of opinion, we decided to use an odd number of seven evaluators in the two types of analysis.

For the validation of the application content, professional expertise with at least two years of experience in TB were defined as inclusion criteria. Because the diagnosis of LTBI is carried out in primary care, secondary, and tertiary references^8^, we decided to include health professionals who worked in different TB care services regardless of their training area.

Currently, the Ministry of Health^10^ recommends that the prescription of treatment is the responsibility of the medical staff, and the nurse is responsible for investigating the LTBI, discussing, and providing support in establishing the diagnosis and monitoring the treatment. TB programs in Brazil have a greater number of nursing professionals, and they are the majority of professionals who coordinate these services. In this way, it is justified that the number of professionals with nursing training is greater than that for medical doctors in relation to the evaluation of the application.

A link was sent by mail inviting the professionals to access the Informed Consent Form (FICF) through “Google Forms Application” and to the study questionnaires (characterization of professional participants and content validation). The content validation questionnaire refers to an application with answers presented on a Likert scale: 1 (strongly disagree), 2 (disagree), 3 (neither agree nor disagree), 4 (agree), and 5 (strongly agree).

In this validation questionnaire, the professionals chose the option they considered most appropriate among the answers, if the option selected was “disagree”, it was necessary to justify the reason for disagreement using the criteria as follows: usefulness/relevance (1), consistency (2), clarity (3), objectivity (4), simplicity (5), executable (6), updating (7), vocabulary (8), precision (9), and institutional sequence of topics (10)^9^ ^,^ ^11^.

The content validity index (CVI) was applied in the first and second versions of the application's questionnaire content. As the number of evaluators was above six, and in order to stipulate an acceptable agreement rate among the professionals, the recommended value of at least 0.80 was established to serve as a decision criterion for item acceptance^12^.

The items that were suggested to be changed or eliminated in the first version were considered, and after the change, they were sent again to the professionals so that we could have the final content validation feedback.

Content validation semantic analysis was also performed. The objective of this stage was to verify whether the items present in the same instrument that the professionals evaluated were understandable for the target audience, who were patients with LTBI. The target audience was also able to recommend modifications deemed necessary to any element of the application. This study was carried out at a health unit located in the city of Vila Velha, Espírito Santo, Brazil. Interested patients participating in the research signed the printed FICF. The researcher completed the content validation questionnaire (the final version approved by the professionals) according to the patient's answers.

This study was approved by the Research Ethics Committee of the Health Sciences Center of the Federal University of Espírito Santo under CAAE No. 88226218.0.1001.5060. In line with Resolution 466/2012 of the National Health Council, all requirements for the protection of participants in scientific research involving humans were met.

The data obtained in the first and second versions of the evaluation of the application content by the professionals and the analysis by the target audience were entered into the BioEstat program, version 5.3, and analyzed using descriptive statistics.

A conceptual map was created to assist in the production of the application content. Using the definition of the conceptual map, a software representation with user-related interfaces was created. The first version of the application screen was produced; in this first version, the research group observed the need to include more pictures instead of text, replace images, improve the layout of the patient's medication schedule and the topic “How am I feeling?”. After making these changes, the second version of the application screen was generated in the presence of more images and less text. Images of the first and second versions of the application are shown in Figure 1.

**FIGURE 1:**
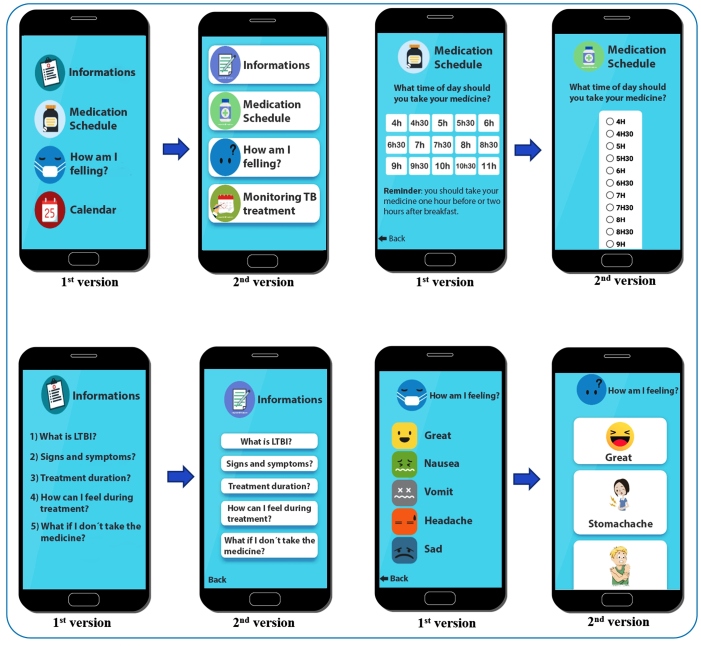
First and second versions of the "My latent tuberculosis treatment" application screens.

Of the seven health professionals who evaluated the application content, 71.4% were nursing graduates and 28.6% were medical doctors; these professionals worked in health secretariats, health units, hospitals, and the Ministry of Health. All participants had more than seven years of training and were from different cities. Table 1 presents the first version of the items related to the application content evaluated by these professionals as well as their suggestions. Among the 20 items evaluated by the professionals, 10 presented a CVI > 0.80, while Items 1, 2, 4, 6, 7, 8, 11, 13, 14, and 19 were reformulated based on their suggestions.

**TABLE 1:** Judgment of the professionals from different cities about the app content, Brazil, 2020.

Evaluated items	Judgment (%)					CVI	Evaluated requirements	Suggestions
	1*	2*	3*	4*	5*			
1.The name of the application will be “*Tratamento Virtualmente Observado da Tuberculose (TVO-TB)*”. Do you think this name is appropriate?	28.6	14.3	28.6	-	28.6	0.25	Usefulness	Change to preventive treatment or tuberculosis - monitoring.
							Consistency	
							Precision	
2.Will this app name be able to draw the attention of patients with LTBI?	28.6	14.3	28.6	-	28.6	0.25	Usefulness	Change to “*Meu tratamento ILTB*” or “Prevenindo TB”.
							Consistency	
							Clarity	
							Vocabulary	
3.The app will have a space for information about LTBI. Do you consider this space adequate?	-	-	-	14.3	85.7	1	-	-
4.The space that will have information about LTBI will be in the form of conversation balloons with a professional talking to the patient. Do you think this will be the best way to show this space?	-	14.3	14.3	57.1	14.3	0.75	Usefulness	-
5.Do you consider the question: “What is LTBI?” important within the app?	-	-	-	14.3	85.7	1	-	-
6.The question “What is LTBI?” will have the answer: “LTBI is Latent Tuberculosis Infection. Latent is that it is not manifesting, or that it is asleep. LTBI occurs when a person is contaminated by a bacteria called *Mycobacterium tuberculosis*. Look at what this bacterium looks like (image of the bacterium). It does not mean that everyone who is contaminated by the bacteria will get sick with the active form of Tuberculosis. In general, infected people remain healthy for many years, without transmitting the bacteria, and with partial immunity to the disease”. How do you rate this answer?	-	-	28.6	14.3	57.1	0.75	Objectivity	Include the information: “If left untreated, the disease can appear in its most severe form”.
7.You consider the question: “Which are the signs and symptoms of LTBI?” important within the app?	14.3	14.3	-	14.3	57.1	0.75	Clarity	Change to affirmative “Which are the signs and symptoms?”
8.The question “Which are the signs and symptoms of LTBI?” will have the following answer: “In most cases of people with LTBI the bacteria is dormant and, thus, it does not progress or cause illness. Then the patient asks the question: But how will I know if I have LTBI? and the professional will answer: When a specific exam is performed by a health professional”. How do you rate this answer?	-	28.6	14.3	28.6	28.6	0.62	Clarity and Precision	Change to affirmative “Which are the signs and symptoms?”
9.You consider the question: “What is the treatment time?” important within the app?	-	-	-	14.3	85.7	1	-	-
10.The question “Which is the treatment time?” will have the following answer: “When you are treated with the Isoniazid antibiotic, the number of doses of the drug taken, not just the length of treatment, is most important. Depending on the amount of dose your doctor gives, the treatment can last from 6 to 9 months, or even 12 months”. How do you rate this answer?	-	14.3	-	57.1	28.6	0.87	-	-
11.Within the space “Which is the treatment time?” there will be a continuation of a conversation between the patient and the professional in which the patient reports: “But if I'm not feeling anything, why am I going to take medication?” then the professional answers: “You will take the medication so that LTBI does not turn into active TB”. How do you rate this answer?	-	14.3	14.3	42.8	28.6	0.75	Vocabulary	Include “There are benefits to treating at this moment, reducing the chances of getting sick from TB”.
12.You consider the question: “What can I feel during treatment?” important?	-	-	-	14.3	85.7	0.87	-	-
13.The question “What can I feel during treatment?” will have the following answer: “You will hardly experience any of these signs and symptoms, but if it happens you may present: Nausea and vomiting, stomach pain, itching, red rash on the skin, joint pain, hands and feet may have weakness or numbness and pain due to damage to these nerves, headache and change of behavior, fever”. All these signs and symptoms will be accompanied by images and captions for ease of understanding. How do you rate this answer?	-	28.6	-	14.3	57.1	0.75	Usefulness and Consistency	Include hepatotoxicity. Replace the word “nausea” with “sickness”.
14.Within this topic “What can I feel during treatment?” will have the information of the professional: “If you have one of these signs (rash, delusions, hallucinations and seizures), contact your health unit immediately and don't forget to report it in the app”. Do you think this information is adequate?	-	-	14.3	28.6	42.8	0.75	Clarity	Replace the term “eruption” with “spots”.
15.Do you consider the following question important: “What happens if I don't take the medication?”	-	-	-	14.3	71.4	1	-	-
16.The question “What happens if I don't take the medication?” will have the following answer: “If you don't take your medication at the right times or even decide not to follow your treatment anymore, you could develop TB, and you know what's worse? Maybe this antibiotic you used this time, it has a great chance of not helping you in the other treatment”. And the patient asks: “So what should I do?” The professional answers: “The ideal is for you to do all your treatment, we are here to help you on this mission, we need you, your health is very important to us”.	14.3	-	-	28.6	57.1	0.87	-	-
17.The app will have a space for the “medication time”, with the following question: “What time do you want to take the medication?”. Do you think this space is important?	-	-	14.3	-	85.7	0.87	-	-
18.The app will have a space where the patient will report what they are feeling. In this space there will be pictures and legends identifying what the patient may feel during their treatment period. For example: When referring to itch, there will be an image of a person scratching themselves and below it will have the word itch written. Do you think it's important?	-	-	-	28.6	71.4	1	-	-
19.The app will have an option called calendar, this space will have information about the initiation, end and current day of treatment. Do you think the word “Calendar” would be ideal to identify this information?	-	14.3	-	57.1	28.6	0.75	Vocabulary	Replace calendar with “Treatment period”.
20.The app will have a voice command that will speak what the patient is reading within the app. Do you consider this information important?	-	-	14.3	-	85.7	0.87	-	-

Caption: 1*I totally disagree; 2*Disagree; 3*I neither agree nor disagree; 4*I agree; 5*I totally agree; CVI (Content Validity Index); Standard Deviation: 0.21.

In the first version of the application content analysis (Table 1), the changes suggested by the professionals were implemented, producing the second version of the application content, as shown in Table 2. We observed that of the 10 items evaluated, only items 1 and 2, referring to the application's name, had a CVI below 0.80. Therefore, we decided not to change these two items and checked the target audience's assessment.

**TABLE 2: Final:** opinion of the professionals about the items with CVI < 0.80, Brazil, 2020.

Items without changes	Reformulated items	CVI of the reformulated items
1.The name of the application will be “*Tratamento Virtualmente Observado da Tuberculose (TVO-TB)*”. Do you think this name is appropriate?	The application name will be “*Meu tratamento ILTB*”. Do you think this name is appropriate?	0.71
2.Will this app name be able to draw the attention of patients with LTBI?	This application name will be able to draw the attention of patients with latent tuberculosis infection (LTBI)?	0.71
4.The space that will have information about LTBI will be in the form of conversation balloons with a professional talking to the patient. Do you think this will be the best way to show this space?	Within the application there will be an “information” menu option; in this part, the patient will have access to important information about LTBI. All this information will be presented with conversation balloons with a professional talking to the patient. Do you think this will be the best way to show this information?	0.86
6.The question “What is LTBI?” will have the following answer: “LTBI is Latent Tuberculosis Infection. Latent is that it is not manifesting, or that it is asleep. LTBI occurs when a person is contaminated by a bacteria called *Mycobacterium tuberculosis*. Look at what this bacterium looks like (image of the bacterium). It does not mean that everyone who is contaminated by the bacteria will get sick with the active form of TB. In general, infected people remain healthy for many years, without transmitting the bacteria, and with partial immunity to the disease”. How do you rate this answer?	The question “What is LTBI?” will have the following answer: “LTBI is Latent Tuberculosis Infection. Latent is that it is not manifesting, or that it is asleep. LTBI occurs when a person is contaminated by a bacteria called *Mycobacterium tuberculosis*. Look at what this bacterium looks like (image of the bacterium). It does not mean that everyone who is contaminated by the bacteria will get sick with the active form of TB. In general, infected people remain healthy for many years, without transmitting the bacteria, and with partial immunity to the disease”. It is noteworthy that, if left untreated, the disease can appear and even more severely. How do you rate this answer?	0.86
7.You consider the question: “Which are the signs and symptoms of LTBI?” important within the app?	Do you consider the statement important: “Signs and Symptoms of LTBI” important within the app?	0.86
8.The question “Which are the signs and symptoms of LTBI?” will have the following answer: “In most cases of people with LTBI the bacteria is dormant and, thus, it does not progress or cause illness. Then the patient asks the question: But how will I know if I have LTBI? and the professional will answer: When a specific exam is performed by a health professional”. How do you rate this answer?	The statement “LTBI signs and symptoms” will have the following content: “In most cases of people with LTBI the bacteria is dormant and, thus, it does not progress or cause illness. Then the patient asks the question: But how will I know if I have LTBI? and the professional will answer: When a specific exam is performed by a health professional”. How do you rate this answer?	0.86
11.Within the space “Which is the treatment time?” there will be a continuation of a conversation between the patient and the professional in which the patient reports: “But if I'm not feeling anything, why am I going to take medication?” then the professional answers: “You will take the medication so that LTBI does not turn into active TB”. How do you rate this answer?	Within the information menu there will be the question “What is the treatment time?”, with a continuation of a conversation between the patient and the professional in which the patient reports: “But if I'm not feeling anything, why am I going to take medication?” and the professional will answer: “You will take your medication, because there are benefits in treating it at this moment, reducing the chances of getting sick from TB. How do you rate this answer?	0.86
13.The question “What can I feel during treatment?” will have the following answer: “You will hardly experience any of these signs and symptoms, but if it happens you may present: Nausea and vomiting, stomach pain, itching, red rash on the skin, joint pain, hands and feet may have weakness or numbness and pain due to damage to these nerves, headache and change of behavior, fever”. All these signs and symptoms will be accompanied by images and captions for ease of understanding. How do you rate this answer?	The question “What can I feel during treatment?” will have the following answer: “You will hardly experience any of these signs and symptoms, but if it happens you may present: Nausea and vomiting, stomach pain, itching, red rash on the skin, joint pain, hands and feet may have weakness or numbness and pain due to damage to these nerves, headache and change in behavior, fever, and hepatotoxicity (it is liver damage that can be caused by the medication)”. All these signs and symptoms will be accompanied by images and captions for ease of understanding. How do you rate this answer?	0.86
14.Within this topic “What can I feel during treatment?” will have the information of the professional: “If you have one of these signs (rash, delusions, hallucinations and seizures), contact your health unit immediately and don't forget to report it in the app”. Do you think this information is adequate?	Also within this topic “What can I feel during treatment?” will have the information of the professional speaking: “If you have one of these signs below (skin patches, delusions, hallucinations, seizures), immediately contact your health unit and don't forget to report it in the app”. Do you think this information is adequate?	0.86
19.The app will have an option called calendar, this space will have information about the initiation, end and current day of treatment. Do you think the word “Calendar” would be ideal to identify this information?	The application will have a “Treatment period” option with information on the initiation, end and current day of treatment. Do you think the term “Treatment Period” would be ideal to identify this information?	1

Of the seven patients who evaluated the content of the app, five (71.4%) were between 30 and 36 years of age and two (28.6%) were over 50 years old; four (57.1%) were male and three (42.9%) were female; five (71.4%) did not complete any level of education, one (14.3%) had completed elementary education, four (57.1%) completed high school, and two (28.6%) had higher education.

It was observed that the patients did not adapt to the name of the application due to the acronym LTBI; most of them stated that it was not possible to establish a relationship between LTBI and latent tuberculosis and that this name was not appealing. Therefore, the application name was defined as “My latent tuberculosis treatment”.

Our application proved to be a tool capable of meeting the needs of patients, as it allows them to access information about the initiation, end, and current day of treatment, allowing them to choose the medication time, in addition to having a voice support that allows for social inclusion. It is important to emphasize that it is a mobile health application; it uses mobile technology to help patients with LTBI adhere to treatment, in addition to providing information about the disease. Having adequate assistance from professionals, in addition to the help of this application, can contribute to adherence to LTBI treatment.

Access to and use of information and communication technologies (ICT) are related to the growing process of technology in contemporary society. To develop an application, it is important to understand the needs of its users^7^.

Applications aimed at health have the ability to reach heterogeneous populations, and are mainly used by middle-aged and older adults. Therefore, it is important for the application to have an uncomplicated, comprehensible language^13^.

The professionals evaluated the first version of the questionnaire, composed of twenty items referring to the content of the application. Ten items were reformulated by the professionals witch made the necessary considerations based on the evaluated requirements. The main changes in relation to the content presented were in relation to “utility, consistency, clarity, objectivity, vocabulary and precision”.

After the changes made for these ten items, a new questionnaire was produced and the professionals evaluated the questionnaire again. Only two itens among ten were suggested changes, related to the application name. After all modifications suggested by the professionals, the patients had access to the questionnaire final version related to the application content.

In the second stage of the study, validation of the application content was conducted with the patients, in which participant education ranged from incomplete elementary education to postgraduate studies, and the changes reported most frequently by these patients were “clarity and simplicity.” It is important that the application content is understood by its target audience, since the risk group includes the most varied types of patients, such as older adults, illicit drug users, recent immigrants from countries with a high TB incidence, people with diabetes, as well as healthcare professionals^14^.

Digital technologies provide opportunities to address the challenges of health systems. This study is a digital health intervention with components that contribute to its implementation, according to the guidelines published by the WHO in 2019¹, such as validated health content and ICT systems that contribute to the strengthening of the health system.

A systematic review showed that digital health interventions are being increasingly used to assist in the treatment of TB and that they can improve medication adherence and patient outcomes^15^. Our study sought to focuses on patients with LTBI using technology.

Although there is a great interest in ICT, some limitations need to be highlighted: the user can find it difficult to understand digital technology and information, and not all individuals have access to a mobile device, especially those of lowest socioeconomic status^14^. 

It is worth noting that content validation excludes drawings and figures that facilitate understanding. It is understood that these items will be evaluated at another time in the study, which is the face validation of the application.

This study developed and validated the application of *My latent tuberculosis treatment*. The patient will have access to an easy-to-understand tool that will help and facilitate adherence throughout treatment. The participation of health professionals and patients was crucial to improve the application language, with the “clarity” domain being the one that needed the most reformulation.
